# The responses of neural stem cells to the level of GSK-3 depend on the tissue of origin

**DOI:** 10.1242/bio.20131941

**Published:** 2013-06-20

**Authors:** Tamara Holowacz, Tania O. Alexson, Brenda L. Coles, Bradley W. Doble, Kevin F. Kelly, James R. Woodgett, Derek Van Der Kooy

**Affiliations:** 1 Terrence Donnelly Centre for Cellular and Biomolecular Research, University of Toronto, Toronto, ON M5S 3E1, Canada; 2 Stem Cell and Cancer Research Institute, McMaster University, Hamilton, ON L8N 3Z5, Canada; 3 Samuel Lunenfeld Research Institute, Mount Sinai Hospital, Toronto, ON M5G 1X5, Canada

**Keywords:** Neural stem cells, Wnt signaling, ES cells, Neurosphere assay

## Abstract

Neural stem cells (NSCs) can be obtained from a variety of sources, but not all NSCs exhibit the same characteristics. We have examined how the level of glycogen synthase kinase-3 activity regulates NSCs obtained from different sources: the mouse embryonic striatum, embryonic hippocampus, and mouse ES cells. Growth of striatal NSCs is enhanced by mild inhibition of GSK-3 but not by strong inhibition that is accompanied by Wnt/TCF transcriptional activation. In contrast, the growth of hippocampal NSCs is enhanced by both mild inhibition of GSK-3 as well as stronger inhibition. Active Wnt/TCF signaling, which occurs normally in the embryonic hippocampus, is required for growth of neural stem and progenitor cells. In the embryonic striatal germinal zone, however, TCF signaling is normally absent and its activation inhibits growth of NSCs from this region. Using a genetic model for progressive loss of GSK-3, we find that primitive ES cell-derived NSCs resemble striatal NSCs. That is, partial loss of GSK-3 alleles leads to an increase in NSCs while complete ablation of GSK-3, and activation of TCF-signaling, leads to their decline. Furthermore, expression of dominant negative TCF-4 in the GSK-3-null background was effective in blocking expression of Wnt-response genes and was also able to rescue neuronal gene expression. These results reveal that GSK-3 regulates NSCs by divergent pathways depending on the tissue of origin. The responses of these neural precursor cells may be contingent on baseline Wnt/TCF signaling occurring in a particular tissue.

## Introduction

Neural stem cells (NSCs), which give rise to all neuronal and glial progenitors of the nervous system, are present throughout development, from embryo to adult (Seaberg and van der Kooy, 2003; Tropepe et al., 1999). Much attention has been given in recent years to the molecular pathways regulating their generation and homeostasis. It is hoped that advances in this line of inquiry will lead to technologies for deriving NSCs from various tissue sources, including embryonic stem (ES) cells. Glycogen synthase kinase-3 (GSK-3) is a serine–threonine kinase that modulates the function of a diverse array of intracellular pathways (Hur and Zhou, 2010; Medina and Wandosell, 2011). Inhibition of GSK-3 has been associated with maintenance of the pluripotent state in ES cells (Miki et al., 2011; Sato et al., 2004). Here we examine the role of GSK-3 in the regulation of NSCs obtained from a variety of sources.

Single NSCs can be isolated from the adult or embryonic brain, and subsequently expanded *in vitro* to form clonal floating spheres, called neurospheres (Tropepe et al., 1999). When a neurosphere is dissociated it can be passaged clonally numerous times. The number of resulting clonal spheres indicates the number of NSCs that were contained in the original population and demonstrates the ability of the NSCs to undergo self-renewal. It should be noted, however, that the vast majority of neural precursor cells in a clonal neurosphere are neural progenitor cells (Morshead et al., 1994), which have decreased passaging ability and limited self-renewal. The number of neural progenitor cells can be correlated with sphere diameter. The progeny of these neural progenitors can be induced to differentiate into neurons and glia both *in vitro* and *in vivo* (Coles-Takabe et al., 2008; Reynolds et al., 1992; Seaberg and van der Kooy, 2003).

Since the first reports of a NSC in the adult forebrain lateral ventricles (Morshead et al., 1994; Reynolds et al., 1992), several other populations of NSCs have been described. An early primitive population (pNSCs) can be derived from undifferentiated ES cells or from epiblast and neurula stage (E5.5–E8.5) mouse presumptive neurectoderm (Hitoshi et al., 2004). Definitive NSCs (dNSCs) can be isolated from the brain after E8.5 and persist into adulthood. pNSCs are LIF dependent, while dNSCs require only FGF or EGF (not LIF) for their proliferation (Hitoshi et al., 2004). Furthermore, regional differences in NSC behavior have been described (Seaberg et al., 2005; Seaberg and van der Kooy, 2002): From embryonic development to adulthood, the anterior lateral ventricle contains NSCs that robustly display the stem cell characteristics of self-renewal and multipotentiality. In contrast, the hippocampus contains neural precursors that possess stem cell characteristics at early embryonic stages. Two groups have shown that some of these cells are able to retain multipotentiality *in vivo* throughout the life of the mouse (Bonaguidi et al., 2011; Mira et al., 2010). However, we have shown that they do not retain multipotentiality *in vitro* or the ability to self-renew into adult stages (Clarke and van der Kooy, 2011; Seaberg et al., 2005; Seaberg and van der Kooy, 2002).

The role of GSK-3/Wnt signaling in the regulation of NSCs appears to be both complex and controversial. In addition to its long-established role in regulating metabolism via glycogen synthase, GSK-3 also controls many cellular events involving cytoskeletal proteins, transcription factors, cell survival and cell cycle machinery. Its role in the “canonical” Wnt pathway has also been widely studied (Doble and Woodgett, 2003). In resting cells, GSK-3 forms a complex with Axin, APC, and β-catenin within which GSK-3 phosphorylates β-catenin and targets it for degradation. Following binding of secreted Wnt molecules to the Frizzled and LRP6 co-receptors, the GSK-3/Axin/APC complex becomes recruited to this receptor and GSK-3 phosphorylation of β-catenin is inhibited (Zeng et al., 2005). As a result, cytoplasmic β-catenin levels rise and some of this β-catenin translocates to the nucleus, where it can associate with LEF/TCF (Lymphoid enhancing factor/T-cell factor) transcription factors and activate gene transcription (Clevers, 2006). GSK-3 has also been implicated in the control of other signaling pathways such as the Notch, receptor tyrosine kinase pathways (like insulin, IGF-1, FGF) and the hedgehog pathway (reviewed by Kim and Snider, 2011). The control of insulin/IGF-1 signaling via GSK-3 has features analogous to Wnt signaling in that GSK-3 activity becomes inhibited upon binding of the insulin/IGF-1 to its receptor (Medina and Wandosell, 2011). However, in this case, PKB/Akt is the upstream regulator of GSK-3 activity. Functional segregation of the insulin/IGF and Wnt pathways requires that the sub-cellular pools of GSK-3 committed to each pathway are somehow separated.

Antagonism of Wnt signaling has been implicated in the conversion of ES cells to neuronal progenitors (Aubert et al., 2002). For NSCs derived from the embryonic cortex, overexpression of Wnt can lead to an increase in the number of neurospheres (Viti et al., 2003). *In vivo*, both the conditional overexpression of β-catenin (Chenn and Walsh, 2002) and the conditional knockout of all GSK-3 alleles (Kim et al., 2009) in neural progenitors result in dramatic hyperproliferation of the neuroepithelium. However, other studies suggest that Wnt decreases neurosphere number (Hirabayashi et al., 2004; Kuwahara et al., 2010; Muroyama et al., 2004) and leads to neuronal differentiation (Hirabayashi et al., 2004; Kuwabara et al., 2009; Kuwahara et al., 2010; Munji et al., 2011; Muroyama et al., 2004).

We hypothesized that the level of GSK-3 activity in stem cells is important in determining whether downstream Wnt/TCF signaling targets become activated or not. Here we utilize drug-mediated inhibition of GSK-3 as well as ES cell lines bearing mutations in different alleles of GSK-3 to examine how the level of GSK-3 regulates NSC proliferation, self-renewal and differentiation. Furthermore, using NSCs derived from different sources (i.e. embryonic striatum, embryonic hippocampus and ES cells), we have revealed a diversity of responses to the modulation of GSK-3.

## Results

### The response of neural precursors to GSK-3 inhibition depends on the tissue of origin

**Fig. 1.**
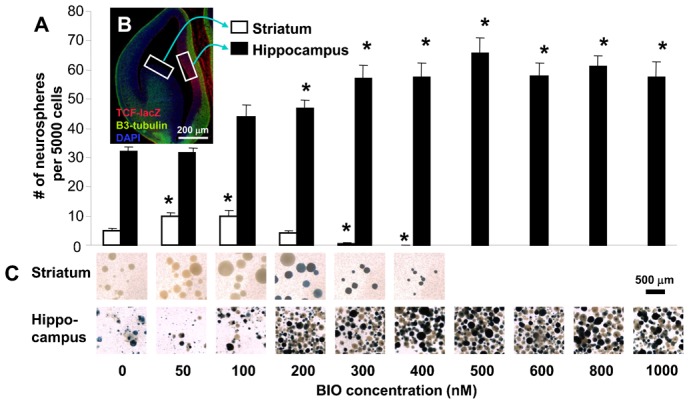
(**A**) The response of neural progenitors to TCF activation depends on the tissue source. When striatal neurosphere cells are exposed to the GSK-3 inhibitor, BIO, the size and number of neurospheres increases at low doses, but they decrease at the higher doses that activate a TCF-lacZ reporter. In contrast, growth of hippocampal neurospheres is improved following TCF activation at much higher doses of BIO. Data presented as mean±S.E.M. Two-way ANOVAs showed significant main effects of tissue type, F_1,120_ = 594.85, *P*<0.05; BIO dose, F_5,120_ = 4.719, *P*<0.05; and interaction, F_5,120_ = 14.055, *P*<0.05. Multiple comparisons using Holm–Sidak post-hoc tests revealed significant differences compared to controls at the doses indicated, *P<0.05. (**B**) Tissue for the neurosphere assay was obtained from the E13.5 ganglionic eminence (i.e. future striatum) and medial pallium (i.e. future hippocampus) as shown. Only the future hippocampus expresses a TCF-lacZ reporter (red). Green, β3-tubulin; blue, DAPI. Scale bar: 200 µm. (**C**) LacZ stain of striatal and hippocampal neurospheres after one week in culture. Positive staining of striatal neurospheres occurs only at higher BIO doses while that of hippocampal neurospheres occurs at all doses. Note that the high density of spheres in C is due to the intentional grouping of many spheres into single wells in preparation for lacZ staining. Spheres were originally cultured at clonal density.

**Fig. 2.**
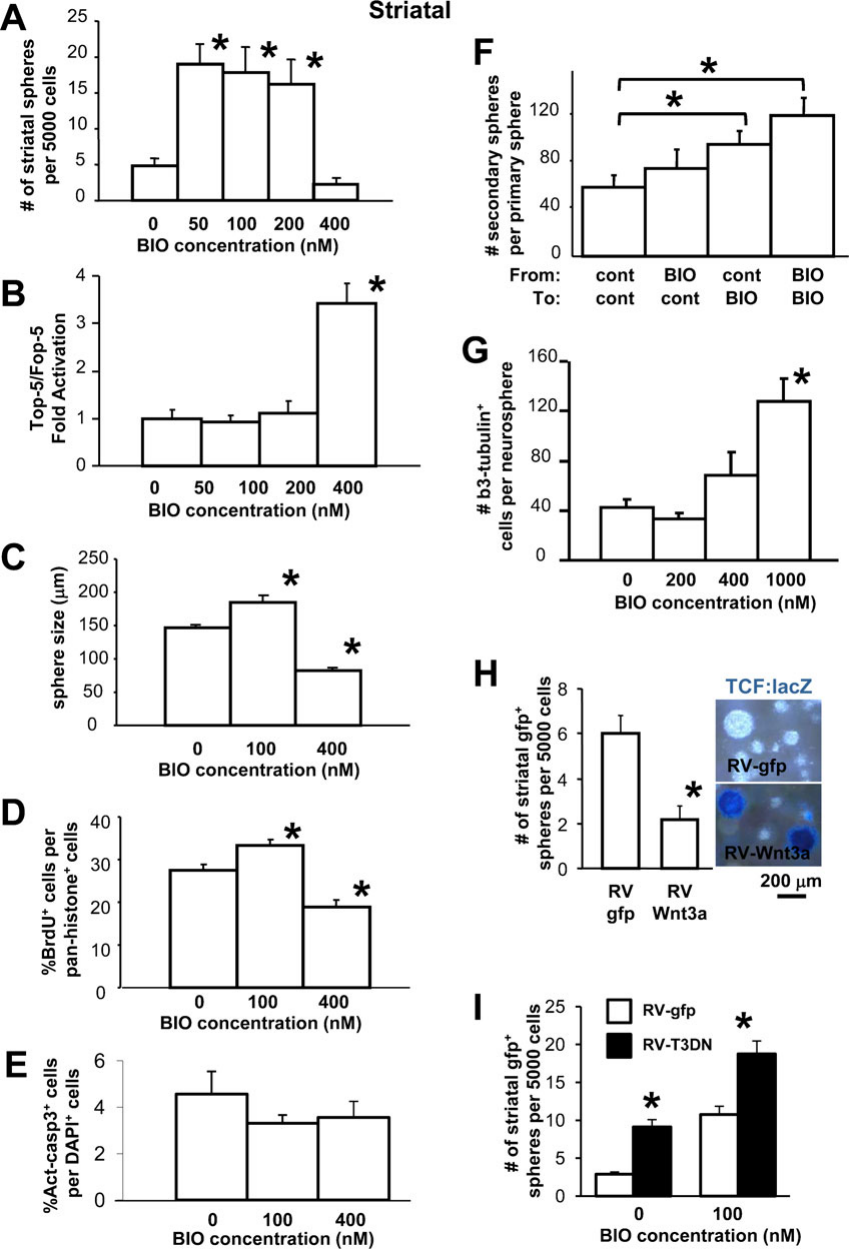
(**A**) When very low sub-micromolar doses of BIO are added to embryonic striatal neurosphere cultures, the number of neurospheres is dramatically increased, indicating an enhancement in growth of neural stem and progenitor cells. At a higher dose of BIO (400 nM), the number of spheres declines. Data presented as mean±S.E.M. One-way ANOVA showed a significant main effect for BIO dose, F_2,25_ = 8.758, *P*<0.05. Multiple comparisons using Holm–Sidak post-hoc test revealed significant differences compared to controls for 50 nM BIO, t_10_ = 3.787, *P<0.05; 100 nM BIO, t_10_ = 3.475, *P<0.05; and 200 nM BIO, t_10_ = 3.029, *P<0.05. (**B**) It was only at the higher dose of BIO (400 nM) that activation of the Top-5-TCF-luciferase reporter was observed. A significant main effect for BIO dose was seen, F_3,32_ = 20.527, *P<0.05. Multiple comparisons revealed a significant difference at 400 nM BIO, t_16_ = 6.426, *P<0.05. (**C**) At a low dose, BIO treated striatal neurospheres were significantly larger than untreated ones. This indicates that stem or progenitor cells within the neurospheres were undergoing either more proliferation or better cell survival. At the higher BIO dose, however, spheres did not grow as large as controls. A significant main effect for BIO dose was seen, F_2,138_ = 40.98, *P*<0.05. Multiple comparisons revealed significant differences compared to control at 100 nM BIO, t_90_ = 5.447, *P<0.05; and 400 nM BIO, t_90_ = 3.219, *P<0.05. (**D**) To monitor proliferation, neurospheres cells were labeled with a 1-hour pulse of BrdU. The number of BrdU^+^ cells was compared to the total number of cells. The number of proliferating cells was increased at the low dose of BIO, but decreased at the higher dose. A significant main effect for BIO dose was seen, F_2,35_ = 28.014, *P*<0.05. Multiple comparisons revealed significant differences compared to control at 100 nM BIO, t_13_ = 4.602,*P<0.05; and 400 nM BIO, t_15_ = 2.911, *P<0.05. (**E**) The number of cells positive for Activated caspase-3 relative to DAPI+ cells was not significantly changed at any dose of BIO. (One-way ANOVA, F_2,21_ = 0.860, *P* = 0.438). (**F**) When individual forebrain neurospheres were clonally passaged, low dose BIO treatment continued to generate more spheres only if present during passaging. This suggests that mild inhibition of GSK-3 enhances neural stem cell proliferation. The lack of a significant increase in neurosphere number after removal of BIO in the secondary passage indicates that stem cell self-renewal is not changed by BIO. A significant main effect for passaging regimen was seen, F_3,143_ = 2.806, *P*<0.05. Multiple comparisons revealed significant differences compared to control for control to BIO medium, t_86_ = 1.993, *P<0.05; and BIO to BIO medium, t_15_ = 2.733, *P<0.05. (**G**) When forebrain neurospheres were cultured in the presence of FGF-containing growth medium very few cells differentiate into neurons. Addition of a high dose of BIO induced expression of β3-tubulin indicative of precocious neuronal differentiation. A significant main effect for BIO dose was seen, F_3,65_ = 10.983, *P*<0.05. Multiple comparisons revealed significant differences compared to control at 1000 nM BIO, t_73_ = 4.784, *P<0.05. (**H**) Retroviral expression of Wnt-3a leads to reduction in the number of primary striatal neurospheres. This suggests that Wnt-3a works like the higher doses of BIO and does not support growth or survival of the striatal neural stem and progenitor cell population (t_8_ = 3.726, *P<0.05). Right panel: LacZ staining of striatal neurospheres from TCF-lacZ embryos showing expression of reporter (blue) following Wnt-3a expression. Scale bar: 200 µm. (**I**) Retroviral expression of dominant-negative TCF3 was used to inhibit canonical Wnt signaling and resulted in increases in the number of neurospheres in control conditions and with 100 nM BIO. A 2-way ANOVA showed significant main effects for retrovirus (F_1,28_ = 42.436, *P<0.05), BIO dose (F_1,28_ = 60.228, *P<0.05) with no interaction (F_1,28_ = 6.125). Multiple comparison procedures reveal a significant difference (*P<0.05) between retroviruses for control BIO (t_14_ = 4.373) and 100 nM BIO (t_14_ = 5.518).

In order to examine the role of GSK-3 in NSC regulation, we first took advantage of the soluble GSK-3 inhibitor, BIO (6-bromoindirubin-3′-oxime) (Meijer et al., 2003), which could be used over a range of concentrations and therefore allowed us to control the level of GSK-3 inhibition. When very low sub-micromolar doses of BIO are added to embryonic day (E) 13.5 clonal striatal neurosphere cultures, both the number and diameter of neurospheres were increased dramatically, indicating an enhancement in growth of all neural precursor cells (i.e. both neural stem and neural progenitor cells; Fig. 1A,C, Fig. 2A,C). Interestingly, as the dose of BIO was raised, the number and size of striatal spheres declined. To examine downstream TCF activity, we used a TCF-lacZ reporter mouse (Mohamed et al., 2004) and performed Top-5-luciferase reporter assays on neurosphere cells (Barker et al., 2007). It was only at a high dose of BIO (400 nM) that we observed activation of the TCF-lacZ reporter in the striatal spheres (Fig. 1C). Likewise, the Top-5-luciferase reporter was activated only at the highest (400 nM) dose of BIO in these striatal spheres (Fig. 2B).

The number and diameter of embryonic hippocampal neurospheres were also increased following BIO treatment at low doses (Fig. 1C). However, in contrast to the striatal spheres (Fig. 1C, Fig. 2C), increases in neurosphere number and diameter were seen following TCF activation even at very high doses of BIO. Therefore the response of neural stem and progenitor cells to TCF activation appears to depend on the tissue of origin. The medial pallium germinal zone, which corresponds to the anterior hippocampal primordium, expresses TCF-lacZ, while the striatal germinal zone does not (Fig. 1B). This could explain why these two tissues respond differently to TCF activation. Where TCF is already active, neural stem and progenitor cells proliferate in response to continued TCF stimulation.

### Inhibition of GSK-3 without TCF activation leads to proliferation of embryonic striatal neural stem and progenitor cells without affecting stem cell self-renewal

The increase in striatal neurosphere number and diameter following mild inhibition of GSK-3 with BIO could be attributed to enhanced proliferation of both neural stem and progenitor cells or to improved cell survival of both cell types (Fig. 2C). BrdU incorporation, which increased at the low dose of BIO, confirmed an increase in proliferation (Fig. 2D). Since most of the cells in a sphere are already in cycle (Kippin et al., 2005), greater BrdU labeling may indicate that the neural precursor cells have a decreased cell cycle time at the low dose of BIO. We did not see any significant difference in the number of cells expressing activated Caspase-3 following treatment with either a low or high dose of BIO (Fig. 2E) suggesting that cell death was not responsible for the changes in sphere size. Therefore the larger diameter of the low dose BIO-treated striatal neurospheres is likely due to increased cell proliferation of neural precursor cells.

Cell proliferation is fundamental to the maintenance and growth of embryonic tissues, but longer term growth requires that the NSC population is also maintained. To determine whether NSC self-renewal is affected by mild inhibition of GSK-3, we performed clonal passaging of primary striatal spheres that were treated with a low dose of BIO (Fig. 2F). In this assay, single clonally-derived neurosphere colonies are selected, mechanically dissociated, and replated in a single tissue culture well. The number of new clonal neurospheres arising from the original colony was counted following one week of culture. This number represents the number of NSCs that were contained in the original colony, which originated from a single neural stem cell. BIO was removed from the secondary passage medium in order to eliminate the confounding effect of BIO on cell proliferation. The lack of a significant increase in secondary neurosphere number under these conditions indicates that NSC self-renewal is not changed by BIO. More spheres were generated only when BIO treatment was present during secondary passaging. With the presence of BIO in the secondary passaging medium, a greater number of larger spheres developed. These were produced via increased progenitor cell proliferation and not through increased NSC self-renewal (Fig. 2C,D). Therefore, it is the enhancement of proliferation of neural progenitor cells that underlies the effect of mild inhibition of GSK-3.

At a higher concentration of BIO that leads to TCF activation we observed a decrease in cell proliferation of striatal neurosphere cells (Fig. 2D). Previous work has shown that activation of TCF signaling with Wnt induces the expression of neuronal differentiation genes such as neurogenin-1 and NeuroD1 (Hirabayashi et al., 2004; Kuwabara et al., 2009). Hence, we examined the extent of neuronal differentiation following inhibition of GSK-3 in striatal neurospheres that were maintained on an adherent substrate in proliferative growth medium (Fig. 2G). At a low dose of BIO, we did not see any change in the number of differentiating, β3-tubulin-positive neurons. However, when the BIO concentration was increased the number of differentiating neurons increased. When neurospheres are grown in media lacking proliferation factors, but with a 1% concentration of serum, more robust neuronal and glial differentiation can be induced (Tropepe et al., 1999). Under these conditions, and in the presence of BIO, significantly more β3-tubulin-positive neurons differentiated at the expense of GFAP-positive astrocytes and O4-positive oligodendrocytes (supplementary material Fig. S1).

The mechanism of BIO-mediated inhibition of GSK-3 is via binding to its ATP binding site (Meijer et al., 2003). Another way to inhibit the action of GSK-3 is via exposure of cells to extracellular Wnt-3a (Clevers, 2006; Doble and Woodgett, 2003; Zeng et al., 2005). Extracellular Wnt-3a leads to the shifting of GSK-3 away from the axin complex and towards the LRP-6 coreceptor. As a result, GSK-3 stops phosphorylating β-catenin. β-catenin can now accumulate in the cytoplasm, translocate to the nucleus and transactivate TCF-transcription. Exposure of striatal NSCs to a retrovirus expressing Wnt-3a lead to a decrease in the number of neurospheres (Fig. 2H). As expected, Wnt-3a also induced expression of the TCF-lacZ reporter within the neurospheres (Fig. 2H). Therefore Wnt-3a mimics the effect of high dose BIO treatment suggesting that striatal NSCs decline following activation of TCF signaling.

To determine whether TCF signaling is involved in the regulation of striatal neural stem cell growth, we infected neurosphere cells with a retrovirus expressing dominant negative TCF-3 (Molenaar et al., 1996) (Fig. 2I). Inhibiting TCF-3 signaling in this way resulted in an increase in the number of neurospheres in both the absence and presence of a low dose of BIO. This provides further evidence that Wnt/TCF signaling opposes the growth of striatal neural stem and progenitor cells.

### Inhibition of GSK-3, leading to TCF activation, causes increased proliferation of embryonic hippocampal neural stem and progenitor cells

**Fig. 3.**
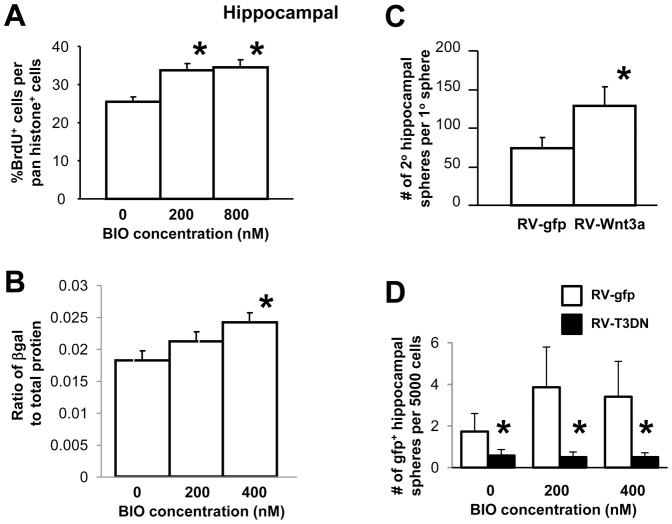
(**A**) To monitor proliferation, E13.5 hippocampus-derived neurosphere cells were labeled with a 1-hour pulse of BrdU. The number of BrdU^+^ cells was compared to the total number of cells. The number of proliferating cells was increased in the presence of BIO in a dose dependent manner. A significant main effect was seen for BIO dose, F_2,57_ = 8.645, *P*<0.05. Holm–Sidak t-test showed significant differences compared to no BIO control; t_38_ = 3.405 for 200 nM BIO, *P<0.05; t_38_ = 3.769 for 400 nM BIO,*P<0.05. (**B**) Increasing amounts of BIO lead to progressive activation of the TCF-lacZ reporter relative to total protein. A significant main effect for BIO dose was seen, F_2,32_ = 3.522, *P<0.05. Multiple comparisons revealed a significant difference at 400 nM BIO, t_16_ = 2.735, *P<0.05. (**C**) Clonal passaging of hippocampal neurospheres is enhanced following retroviral expression of Wnt-3a. Data presented as mean±S.E.M. (t_105_ = 1.999, *P<0.05). (**D**) Retroviral expression of dominant-negative TCF3 was used to inhibit canonical Wnt signaling and resulted in decreases in the number of hippocampal neurospheres. A 2-way ANOVA showed significant main effects (*P<0.05) for retrovirus (F_1,18_ = 79.154), BIO dose (F_2,18_ = 6.363), with an interaction (F_2,28_ = 9.134). Multiple comparison procedures reveal a significant difference (*P<0.05) between retroviruses for control BIO (t_6_ = 3.286), 200 nM BIO (t_6_ = 6.419) and 400 nM BIO (t_6_ = 5.233).

In contrast to E13.5 striatal neurospheres, E13.5 hippocampal neurospheres increased in number and size in response to both mild as well as strong inhibition of GSK-3 (Fig. 1A,C). To determine whether this was due to a change in cell proliferation, we performed BrdU labeling and found that proliferation increased following both a low (200 nM) and high (400 nM) BIO concentration (Fig. 3A). Because Caspase-3 activation was not significantly changed following either high or low dose BIO treatment (data not shown; one-way ANOVA for BIO dose: F_2,11_ = 0.827, *P* = 0.468), we surmise that cell survival was not a factor leading to the increased number of hippocampal neurospheres.

Using the cells from the TCF-lacZ reporter mouse (Mohamed et al., 2004), we monitored the levels of TCF activation in hippocampal neurosphere cells by measuring the level of β-galactosidase relative to total protein following exposure to different concentrations of BIO. Baseline β-galactosidase expression was already high in these spheres compared to striatal neurospheres, but adding BIO significantly increased the levels (Fig. 1C, Fig. 3B).

To test whether activation of TCF in these hippocampal precursor cells via Wnt could mimic the effect of strong inhibition of GSK-3, we exposed these cells to the Wnt-3a-gfp retrovirus and passaged individual neurospheres. Significantly more secondary sphere colonies arose from these neurospheres compared to control, gfp-only-expressing, primary spheres (Fig. 3C). Inhibition of TCF signaling using a retrovirus that expresses dominant negative TCF-3 (Molenaar et al., 1996) resulted in a dramatic decrease in the number of hippocampal neurospheres (Fig. 3D). The addition of BIO did not rescue the hippocampal neurospheres suggesting that TCF-activation mediates the growth of hippocampal neural precursor cells downstream of the inhibition of GSK-3. These results are in direct contrast to those for embryonic striatal spheres (Fig. 2H,I), and are consistent with our observation that Wnt/TCF activation has differential effects on striatal versus hippocampal neural precursor populations.

### A genetic model for progressive loss of GSK-3 also leads to enhanced neurosphere formation

**Fig. 4.**
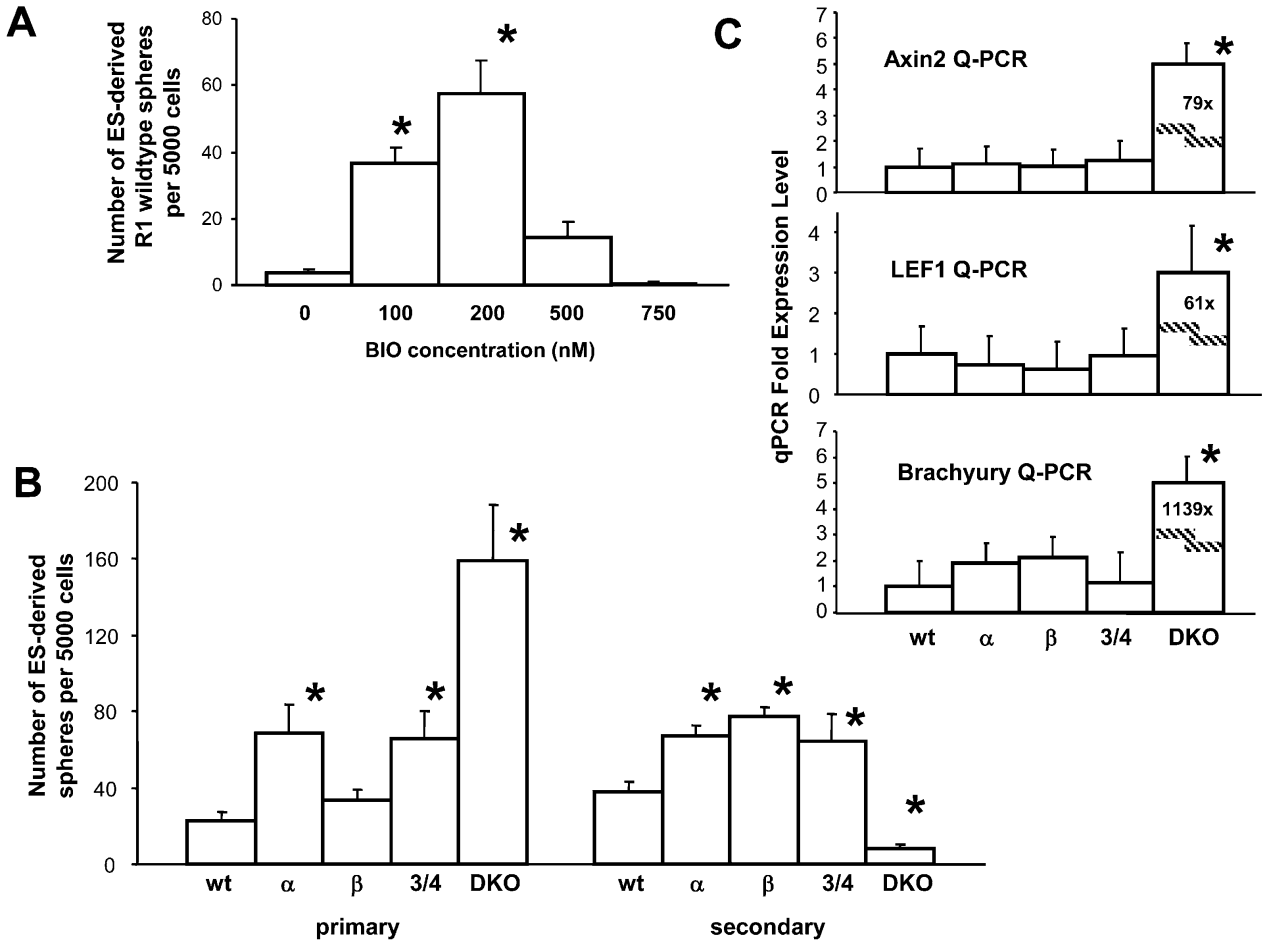
(**A**) ES-cell-derived primitive neurospheres show the same biphasic response to BIO as embryonic striatal neurospheres, but not the increasing dose-dependent response of hippocampal neurosphere cells. Data presented as mean±S.E.M. One-way ANOVA showed significant differences for BIO dose; F_4,23_ = 19.605, *P*<0.05. Post-hoc t-tests showed significant increases compared to no BIO control: 100 nM BIO, t_10_ = 4.277, *P<0.05; and 200 nM BIO, t_10_ = 6.955, *P<0.05). (**B**) Secondary passage neurospheres generated from ES cell lines bearing different genetic levels of GSK-3 also show a biphasic response to the dose of GSK-3. The homozygous loss of either α or β subtypes of GSK-3 or loss of both α and one β subtype (i.e. the “3/4” mutant) results in enhancement of neural stem cells. GSK-3-null (double knockout, DKO) cells generated more neurospheres in primary but not secondary passages. Primary DKO spheres express the mesoderm-specific marker Brachyury (see panel C) and may represent persisting ES cell colonies or embryoid bodies. Upon secondary passaging, DKO spheres are dramatically decreased in number, while the knockout lines with intermediate levels of GSK-3 generate more neurospheres. For primary neurospheres, one-way ANOVA showed a significant effect of genotype, F_4,94_ = 10.402, *P*<0.05. Post-hoc t-tests showed significant increases compared to wildtype for the homozygous α mutant, t_62_ = 2.931, *P<0.05; the ¾ mutant, t_62_ = 2.768, *P<0.05; and the DKO mutant, t_62_ = 5.817, *P<0.05. For secondary neurospheres, one-way ANOVA also showed significant differences for genotype, F_4,94_ = 12.854, *P*<0.05. Post-hoc t-tests showed significant increases compared to wildtype for the homozygous α mutant, t_60_ = 2.726, *P<0.05; the homozygous β mutant, t_60_ = 3.610, *P<0.05; and the DKO mutant, t_60_ = 2.626, *P<0.05). (**C**) Quantitative PCR analysis of secondary neurospheres shows that Wnt-responsive genes are only active in DKO neurospheres. One-way ANOVAs performed for each marker showed significant effect of genotype (F_4,10_ = 2381.74 for Axin2, F_4,10_ = 1083.49 for LEF1, F_4,10_ = 2.7×10^5^ for Brachyury). Post hoc t-tests showed significant differences for the DKO line compared to wildtype for each marker (t_3_ = 77.256 for Axin2, t_3_ = 77.256 for LEF1, t_3_ = 77.256 for Brachyury, *P<0.05).

We found that inhibiting GSK-3 using the chemical inhibitor, BIO, caused enhanced proliferation of hippocampal precursors at both high and low doses (Fig. 1A,B, Fig. 3A), while striatal neurospheres displayed a biphasic response to the dose of BIO (Fig. 1A,B, Fig. 2C,D). That is, E13.5 striatal neural precursors showed increased proliferation at low doses but decreased their proliferation at higher doses. Similar to striatal neurospheres, primitive NSCs, derived clonally from R1 mouse ES cells, also behave biphasically in response to the doses of BIO (Fig. 4A). We employed the canonical Wnt ligand inhibitor, Sfrp2, to see if blocking secreted Wnt affected the number of neurospheres arising from ES-derived primitive NSCs. Sfrp2 increased the number of primitive neurospheres (supplementary material Fig. S2A), suggesting that canonical Wnt signaling in ES cells may impede NSC proliferation.

We also used a genetic model in order to validate our results obtained via drug-mediated inhibition of GSK-3 activity (Doble et al., 2007). Given that there are two GSK-3 genes in mammalian cells (i.e. GSK-3α and GSK-3β), two, three or all four alleles can be ablated in order to control the overall level of GSK-3 activity (Doble et al., 2007). These cell lines are particularly useful for examining the effect of GSK3 dosage since the total amount of GSK3 protein as well as overall GSK3 kinase activity correlates very closely with gene dosage (Doble et al., 2007). Therefore we generated clonal primitive neurospheres from ES cell lines that bear these different genetic levels of GSK-3. The homozygous loss of either α- or β-subtypes of GSK-3, or the loss of both β-subtypes and one α-subtype (i.e. the “3/4” mutant), resulted in an enhancement of NSCs (Fig. 4B). For GSK-3-null (double knockout, DKO) cells, more neurospheres were generated in primary culture (in the presence of LIF) but not secondary passages (in the presence of LIF and FGF). The primary DKO neurospheres may represent persisting ES cell colonies or embryoid bodies since they express very high levels of the mesoderm-specific marker Brachyury (Fig. 4C). Upon secondary passaging, DKO neurospheres were lost, while the knockout lines with intermediate levels of GSK-3 generated more neurospheres than wildtype secondary neurospheres (Fig. 4B). It is likely that secondary passaging promoted further development of the NSCs from the ES cells. Another characteristic of these graded GSK-3 knockout lines consistent with the effects of increasing BIO dose is an increase in secondary sphere diameter for the “intermediate” GSK-3β knockout line, but a decrease in secondary sphere diameter for the full DKO (supplementary material Fig. S2B). Expression of Wnt response genes, Axin-2 and LEF-1, was relatively unchanged in the intermediate GSK-3 knockout secondary neurospheres compared to wildtype controls (Fig. 4C), while in the few enduring secondary DKO spheres, very high expression from these two genes was detected (Fig. 4C). Therefore, the effect of different levels of GSK-3 in these secondary passage neurospheres mimics the biphasic dose effects seen with administration of the GSK-3 inhibitor, BIO. This result supports the hypothesis that complete loss of GSK-3, which leads to activation of TCF-regulated genes, does not support NSC development, while partial loss of GSK-3, as achieved via the loss of some but not all alleles, enhances NSC proliferation.

**Fig. 5.**
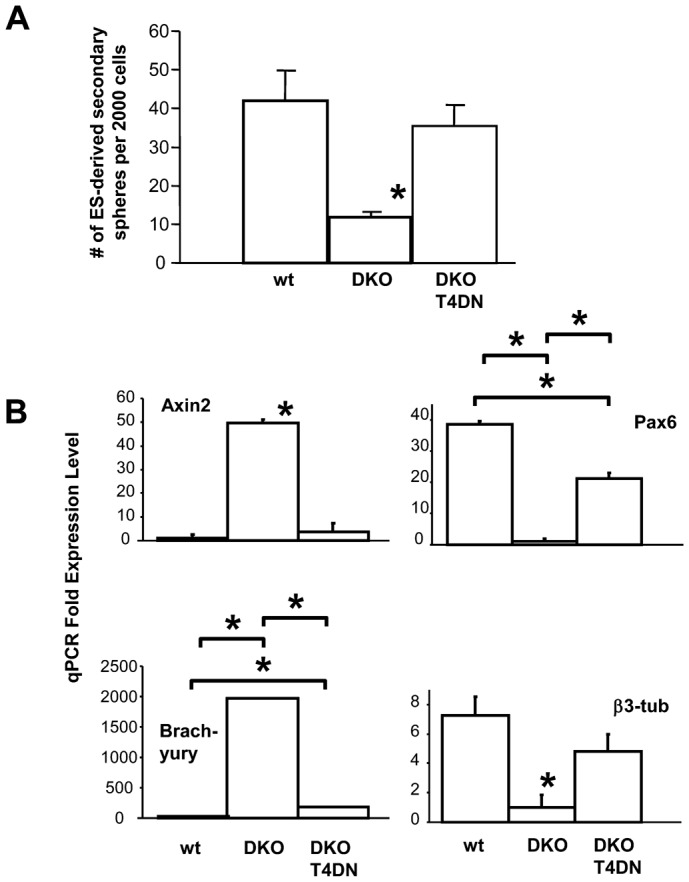
(**A**) TCF signaling is not required for the generation of ES-derived neural stem cells. Loss of all alleles of GSK-3 (DKO) leads to loss of secondary ES-derived neurospheres (because they switch to a mesodermal fate). However, when TCF signaling is blocked in these cells with dominant negative TCF-4 (T4DN), spheres are regained. Data presented as mean±S.E.M. One-way ANOVA showed a significant effect of genotype, F_2,42_ = 9.071. Holm–Sidak t-tests showed significant difference for the DKO line, t_29_ = 3.958, *P<0.05. (**B**) Dominant negative TCF-4 in the GSK-3-DKO background rescues neural progenitors. Quantitative PCR analysis shows that blocking TCF signaling downregulates expression of Wnt response genes Axin2 and Brachyury in GSK-3-DKO ES-derived neurospheres. Dominant negative TCF4 also rescues expression of the neural progenitor marker, Pax6, and the early neural differentiation marker, β3-tubulin. Data represent means±S.E.M. One-way ANOVAs performed for each marker showed significant effect of genotype (F_2,6_ = 1184.05 for Axin2, F_2,6_ = 136.6 for Pax6, F_2,6_ = 5.6×10^5^ for Brachyury, F_2,6_ = 7.999 for β3-tubulin). Multiple comparisons using Holm–Sidak post-hoc t-tests revealed significant differences as indicated, *P<0.05.

Under neurosphere differentiation conditions, all the GSK-3 knockout neurospheres, except the DKO, are able to differentiate into neurons (supplementary material Fig. S2C). For the DKO, spheres did not spread on the substrate and remained spherical indicating a lack of differentiation. Furthermore PCR analysis of the expression of neural differentiation markers, Pax6 and β-3-tubulin, in DKO spheres was considerably reduced compared to wildtype controls (see below and Fig. 5B). Therefore, based on their inability to persist during passaging, their high expression of Brachyury and their inability to differentiate into neurons, we conclude that the DKO colonies seen under neurosphere conditions are not *bona fide* neurospheres but represent a type of ES cell or embryoid body that contains mesoderm.

### TCF activation is not required for the generation of ES-derived NSCs

Loss of all alleles of GSK-3, as achieved in the DKO ES cell line, leads to a loss of secondary clonal spheres, perhaps because they do not achieve a neural fate, but instead maintain a pluripotent fate or switch to a more mesodermal fate (Fig. 4C). These spheres have very high TCF transcriptional activity as indicated by high expression of Wnt/TCF-responsive genes (Fig. 4C). To see if this TCF activity is responsible for the lack of NSC development, we prepared clonal primary primitive spheres from dominant-negative TCF-4 ES cells that were in the DKO background (T4DN). We found that blocking TCF signaling in the absence of GSK-3 rescued growth of secondary neurospheres (Fig. 5A). Expression of Wnt-responsive genes, Axin and Brachyury, while high in the DKO neurospheres, was eliminated following dominant-negative TCF-4 (Fig. 5B), resembling wildtype neurospheres. These results demonstrate that TCF signaling is not required for the generation of primitive NSCs. To confirm that these rescued neurospheres represent *bona fide* NSCs, we also performed quantitative PCR analysis for neural markers. DKO primitive neurospheres lost expression of the neural progenitor marker, Pax6, as well as the early neural differentiation marker, β3-tubulin. Interestingly, when TCF signaling was blocked in the DKO background (T4DN), the expression of these neural markers was regained (Fig. 5B). Therefore loss of GSK-3 only leads to loss of neural stem cells when TCF signaling is activated.

## Discussion

The results presented here show that GSK-3 inhibition has different effects on NSCs depending on the type of NSC under examination (summarized in Table 1). We found that the growth of ES-derived primitive NSCs and striatal definitive NSCs are enhanced by mild inhibition of GSK-3 but not by the strong inhibition that is accompanied by Wnt/TCF activation. In contrast, the growth of hippocampal NSCs is enhanced by both mild inhibition of GSK-3 as well as stronger inhibition that stimulates TCF signaling.

**Table 1.**
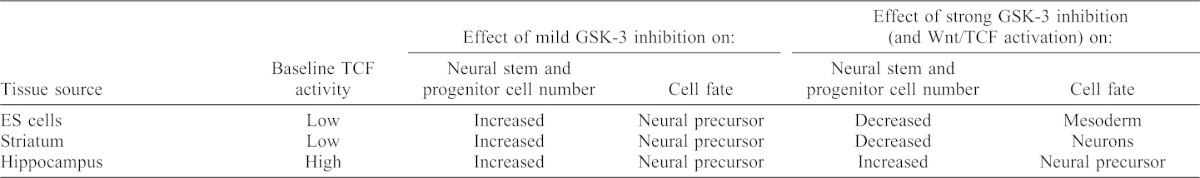
Neural stem and progenitor cells are regulated by GSK-3 and Wnt/TCF signaling.

Inhibition of GSK-3 is frequently used as a way to activate canonical Wnt/TCF-regulated transcription (Doble and Woodgett, 2003). Our results have revealed that embryonic striatal NSCs decline after exposure to Wnt ligand, while hippocampal NSCs are enhanced (Figs 2, 3). In contrast, inhibiting Wnt/TCF activation via dominant negative TCF-3, resulted in the converse circumstance with enhancement of striatal NSCs and decline of hippocampal NSCs (Figs 2, 3). Previous results have shown that Wnt supports pluripotency of ES cells (Yi et al., 2011) and that the switch to a NSC fate appears to involve attenuation of Wnt signaling (Aubert et al., 2002). Consistent with these results, we found that the Wnt inhibitor, Sfrp2, enhanced primitive NSC development from wildtype ES cells (supplementary material Fig. S2). Therefore primitive NSCs appear to be negatively regulated by Wnt, similar to definitive embryonic striatal NSCs. Wnt proteins (ten Berge et al., 2011) and LIF (Williams et al., 1988) are required for maintaining ES cell self-renewal. When Wnt-signaling is blocked, ES cells convert into epiblast stem cells (ten Berge et al., 2011), and subsequently into early neural progenitor cells (Slawny and O'Shea, 2011). But the further differentiation into β3-tubulin positive neurons requires reestablishment of Wnt signaling (Slawny and O'Shea, 2011). Therefore dynamic regulation of Wnt signaling appears to be involved in the lineage of ES cells to mature neurons. In other words, a decrease in Wnt signaling promotes a default neural state, but subsequent to this, increased Wnt signaling is instructive for neuronal differentiation. In contrast, the development of mesoderm from ES cells requires an early Wnt signal (Anton et al., 2007). Hence the observed mesodermal phenotype that was obtained from the DKO GSK-3 ES cell line, (the line which has high TCF activity), upon transfer to serum-free neurosphere medium (Fig. 4C).

Our results revealed more subtle regulation of NSCs by GSK-3 when inhibition of this enzyme was carefully titrated. Partial inhibition of GSK-3 activity, using a drug or as achieved using a genetic model, always enhanced the number of NSCs generated, regardless of the tissue source. But when GSK-3 activity was more strongly inhibited, to levels that lead to activation of TCF-signaling, only the hippocampal neural stem and progenitor cells continued to proliferate (Fig. 3A). In many systems, a cell's response to signals depends on the cell's history leading to a context-dependent interpretation of signals (Iovino and Cavalli, 2011). We found that the baseline TCF activity of embryonic hippocampal neural progenitors was high while that of their striatal counterparts was far lower (Fig. 1). It is likely that this difference underlies the difference in response of these two NSC types to strong GSK-3 inhibition as well as to Wnt ligand exposure. Previous work has shown that cells from the entire embryonic forebrain sorted for expression of an Axin-1-reporter (an alternative TCF-reporter) also display a differential response to Wnt (Kalani et al., 2008). Only the Axin-1-positive cells were able to self-renew in the presence of Wnt. The particular assortment of transcription factors present in chromatin may determine how a cell is poised to respond to upstream cell signals and, upon stimulation, results in a specific cell behavior outcome. For hippocampal NSCs, strong inhibition of GSK-3 accompanied by TCF activation means increased cell proliferation and self-renewal. For striatal NSCs, it means a decline in NSC numbers and neuronal differentiation. And for ES-derived primitive NSCs it also means a decline in neural fates with conversion to the mesodermal lineage (Table 1).

We found that the partial inhibition of GSK-3 appeared to support proliferation of neural stem and progenitor cells regardless of tissue source. Many pathways, other than the canonical Wnt pathway, are also regulated by GSK-3 (Medina and Wandosell, 2011). It is these other pathways that may explain the response to the mild inhibition of GSK-3. Among them, the signaling stimulated by tyrosine kinase receptors, such as Insulin/IGF1, NGF, BDNF, bear a common feature: the inhibition of GSK-3 via phosphorylation of a specific serine residue (and not through the Wnt-mediated GSK-3/Axin/APC complex) (Ng et al., 2009). GSK-3 molecules involved in these pathways may also occupy separate cell compartments with varying permeability and sensitivity to genetic depletion or drug-mediated inhibition.

Recent work has shown that expression of a dominant negative TCF (on the DKO background) supports neural development from ES cells but only when β-catenin levels are significantly knocked down (Kelly et al., 2011). In contrast, our results show that dominant negative TCF4 alone (on the DKO background) was able to support expression of neuronal differentiation markers in primitive ES-derived NSCs (Fig. 5B). One possible reason to explain this difference is our use of the clonal neurosphere assay as opposed to assays employing EBs, teratomas or N2B27 differentiation medium. The neurosphere assay may push cells toward the neural fate better than these other assays and hence prime them to differentiate into neurons without β-catenin knockdown. Secondly, cells of the mesodermal and endodermal germ layers that also develop in these other assay systems may release signals that actively inhibit neuronal differentiation.

The use of NSCs for tissue regeneration therapies requires a full understanding of their regulation. Because of the availability of potential chemical inhibitors, GSK-3 lends itself as a target of drug-mediated modulation. Care, however, must be taken to ensure that only the desired pathways regulated by GSK-3 are affected. Our findings show, first, that the baseline level of GSK-3 activity in different NSC populations is important in controlling neural stem and progenitor proliferation and differentiation, and second, they demonstrate that inhibition of GSK-3 is not always synonymous with downstream Wnt pathway (TCF) activation. Most important, the response of a NSC to GSK-3 can be profoundly affected by the type of tissue from which the NSC was derived.

## Materials and Methods

### Animals and embryonic neurosphere cultures

*TCF-lacZ* mice were generated as described (Mohamed et al., 2004). NSCs were isolated from the lateral ganglionic eminence (future striatum) or medial pallium (future hippocampus) of E13.5 *TCF-lacZ* or *CD1* mouse embryos (Charles River Laboratories) and used for the neurosphere assay (Tropepe et al., 1999). Matings were timed such that midday of the day the vaginal plug was found was considered to be embryonic day E0.5. All animal experiments were carried out according to the protocols approved by the University of Toronto Animal Care Committee.

For the neurosphere cultures derived from embryonic tissues (Tropepe et al., 1999), cells were mechanically dissociated and plated at 10 cells/µl in serum free medium (SFM) containing 10 ng/mL FGF2 and 1 µg/mL heparin. B27 supplement (Invitrogen) was at a 1:50 dilution for hippocampal cultures. Neurospheres (≧100 µm diameter) were counted and diameters measured one week later. BIO (6-bromoindirubin-3′-oxime, EMD Biosciences) was diluted in DMSO at a concentration of 5 mM. It was diluted 1:100 in SFM at room temperature, filter sterilized then diluted further in culture medium to the indicated working concentration. For the luciferase assay used to detect TCF transcriptional activity, mechanically-dissociated neurospheres cells were pelleted by centrifugation and transfected with pRL and Top-5 or Fop-5 (Barker et al., 2007) plasmids using Lipofectamine LTX with Plus Reagent (Invitrogen) according to the manufacturer's instructions. Cells were plated in SFM with growth factors and BIO and luciferase activity was determined the following day using the Dual-Glo Luciferase assay system (Promega).

For single sphere passaging, individual 200–250 µm diameter spheres were selected, mechanically dissociated using a plastic P200 pipette tip and plated in a single well of a 24-well plate. The number of secondary spheres arising from a single primary sphere was counted after one week in culture. For bulk passaging, neurosphere cells were mechanically dissociated and replated at 10 cells/µl in a 24-well plate.

### ES cell-derived primitive neurosphere assay

Primitive neural stem cells were prepared from wildtype and four GSK-knockout ES cell lines (Doble et al., 2007) and from wildtype R1 ES cells as previously described (Tropepe et al., 2001). They were cultured at 10 cells/µl in 24-well plates in SFM with 1000 U/ml LIF. Secondary passage neurosphere cultures were prepared by bulk dissociation using trypsin/EDTA and cultured at 10 cells/µl in SFM with 1000 U/ml LIF, 10 ng/mL FGF and 2 µg/ml heparin. Sfrp-2 (R&D Systems) was added to the culture medium as indicated.

### Quantitative polymerase chain reaction analysis

RNA was isolated from neurosphere cultures using the RNeasy kit (Qiagen). First strand synthesis was carried out using a Superscript III kit (Invitrogen). Taqman qPCR primer and Master Mix (Applied Biosystems) were used according to the manufacturer's directions. Quantitative polymerase chain reaction was performed using a 7900HT Real-Time polymerase chain reaction machine (Applied Biosystems). SDS-2.3 software was used to calculate relative differences in gene expression levels with β-actin used for normalization.

### Detection of *LacZ* expression and BrdU labeling

For *LacZ* staining, intact neurospheres obtained from *TCF-lacZ* embryos were incubated for 1 hour (37°C) in SFM on 6-well plates coated with Matrigel (BD Biosciences) then fixed and stained with β-galactosidase according to Lobe et al. (Lobe et al., 1999). Levels of β-galactosidase following 3 day exposure to BIO were assayed and compared to the level of total protein using the Mammalian β-Galactosidase Assay and Pierce BCA Protein Assay Kits (both from Thermo Scientific) according to the manufacturer's protocols.

For analysis of cell proliferation by means of BrdU-labeling, dissociated neurosphere cells were plated onto fibronectin-coated 24-well plates in SFM containing FGF and heparin at a density of 10 cells per µL and cultured for 4 days. Cells were exposed to a one hour pulse of bromodeoxyuridine (BrdU, 0.6 µM) and then fixed with paraformaldehyde. To detect BrdU, cells were incubated in 4N HCl for 30 minutes and immunostained using mouse anti-pan histone (Chemicon #MAB3422, 1:500) and rat anti-BrdU (Abcam #ab6326, 1:500) antibodies. Secondary antibodies were used at a 1:200 dilution (Jackson Immunochemicals: FITC-donkey-anti-rat IgG #712-095-153 and TRITC-goat-anti-mouse IgG #115-025-146).

### Retroviral infection of neurosphere cells

Mouse *Wnt-3a* (Kengaku et al., 1998) and *Xenopus*-dominant negative TCF3 (Molenaar et al., 1996) expressing retroviruses (*pMXIE-Wnt3a-gfp* and *pMXIE-XT3DN-gfp*) were prepared as described (Holowacz et al., 2011). *pMXIE-gfp* (empty vector) was used as a control. For infection, NSCs were isolated from E14 embryonic striatum or hippocampus of *TCF-lacZ* mice, plated at 100 cells/µl and infected overnight with 2×10^5^ viral particles/ml in 10 ml flasks. The following day, cells were gently triturated using a flame polished glass pipette and plated at a 1:5 dilution in 40 ml flasks. Because hippocampal cells typically adhered to each other at this stage, complete mechanical dissociation of both hippocampal and striatal cells was repeated 5 days later and cells were then replated at 10 cells/µl in 24-well plates and in flasks. The number of gfp^+^ neurospheres (≧100 µm diameter) and/or degree of *lacZ* staining (Lobe et al., 1999) was assessed after one week of culture.

### Differentiation assays and immunocytochemistry

Differentiation of neurospheres was induced by adhering individual spheres onto Matrigel coated 48-well plates and culturing in SFM with 1% fetal calf serum. After one week, colonies were fixed with 4% paraformaldehyde in phosphate buffered saline (PBS, pH 7) and immunostaining was carried out as described previously (Holowacz et al., 2011; Tropepe et al., 1999). Primary antibodies employed in this study are as follows: mouse anti-β3-tubulin (Chemicon #MAB1637, 1:500); rabbit anti-activated Caspase3 (Promega #G748, 1:500); rabbit anti-GFAP (Wako #ZO334, 1:1000); mouse anti-O4 (Chemicon #MAB345, 1:500). Anti-rabbit or anti-mouse Alexa Fluor 488 or 568 nm secondary antibodies were obtained from Molecular Probes and used at a dilution of 1:400. Nuclei were visualized by incubating cells or slides for 20 min with 0.1 µg/mL DAPI (Sigma) in PBS.

### Statistical analysis

All data are expressed as means±S.E.M. Statistical comparisons were performed using the Sigmastat 3.1 software package. ANOVA or t-tests were used to analyze data as appropriate. Significant ANOVA tests were followed by *post hoc* comparisons of individual means using Holm–Sidak t-tests where appropriate. The level of significance for all comparisons was defined as 2-tailed at *P*<0.05.

## Supplementary Material

Supplementary Material
