# Supplementary material for: The responses of neural stem cells to the level of GSK-3 depend on the tissue of origin
Source: Biol Open. 2013 Jun 20;2(8):812–21. doi: 10.1242/bio.20131941 (PMC3744073; doi:10.1242/bio.20131941)
Supplement: Supplementary Material [file supp_2_8_812__index.html]

The responses of neural stem cells to the level of GSK-3 depend on the tissue of origin — The responses of neural stem cells to the level of GSK-3 depend on the tissue of origin — Supplementary Material 

# The responses of neural stem cells to the level of GSK-3 depend on the tissue of origin

## 

**Files in this Data Supplement:**

- Supplementary Material - Tamara Holowacz et al. doi: 10.1242/bio.20131941
