# Supplementary material for: The responses of neural stem cells to the level of GSK-3 depend on the tissue of origin
Source: Biol Open. 2013 Jun 20;2(8):812–21. doi: 10.1242/bio.20131941 (PMC3744073; doi:10.1242/bio.20131941)
Supplement: Supplementary Material [file supp_bio.20131941_bio.20131941-s1.pdf]

Supplementary Material

Tamara Holowacz et al. doi: 10.1242/bio.20131941

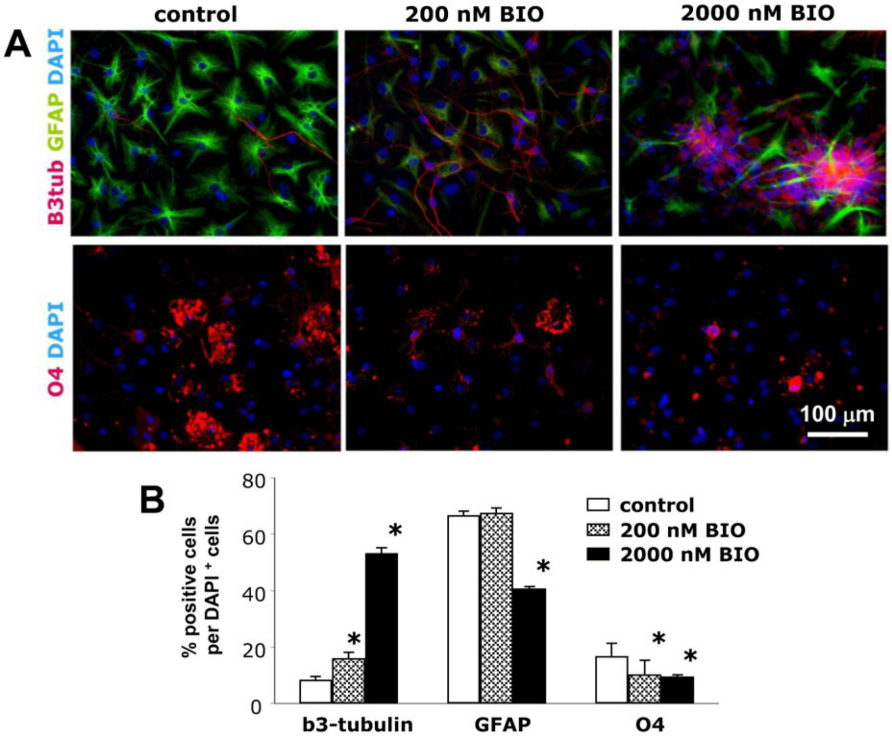

Fig. S1. (**A**) Neurospheres were cultured in differentiation conditions with different concentrations of BIO and expression of neuronal markers was assessed by immunofluorescence. More cells differentiated into neurons at higher BIO concentrations (β3-tubulin, red). Note dense grouping of neurons at the highest concentration of BIO. GFAP stain for astrocytes, green; blue, DAPI stain for DNA. In contrast, fewer oligodendrocytes were seen when the BIO concentration was increased (O4, red). Scale bar: 100 μm. (**B**) Quantization of the results shown in panel A. BIO enhanced neuronal differentiation at the expense of astrocytes and oligodendrocytes. All data expressed as mean±S.E.M. One-way ANOVAs for each cell type show significant differences for BIO concentration; β3tubulin: $F_{2,12}=74.17$, $P<0.05$; GFAP: $F_{2,12}=22.12$, $P<0.05$; O4: $F_{2,12}=12.14$, $P<0.05$. Holm–Sidak t-tests for individual concentrations revealed significant differences as shown, *$P<0.05$.

Biology Open

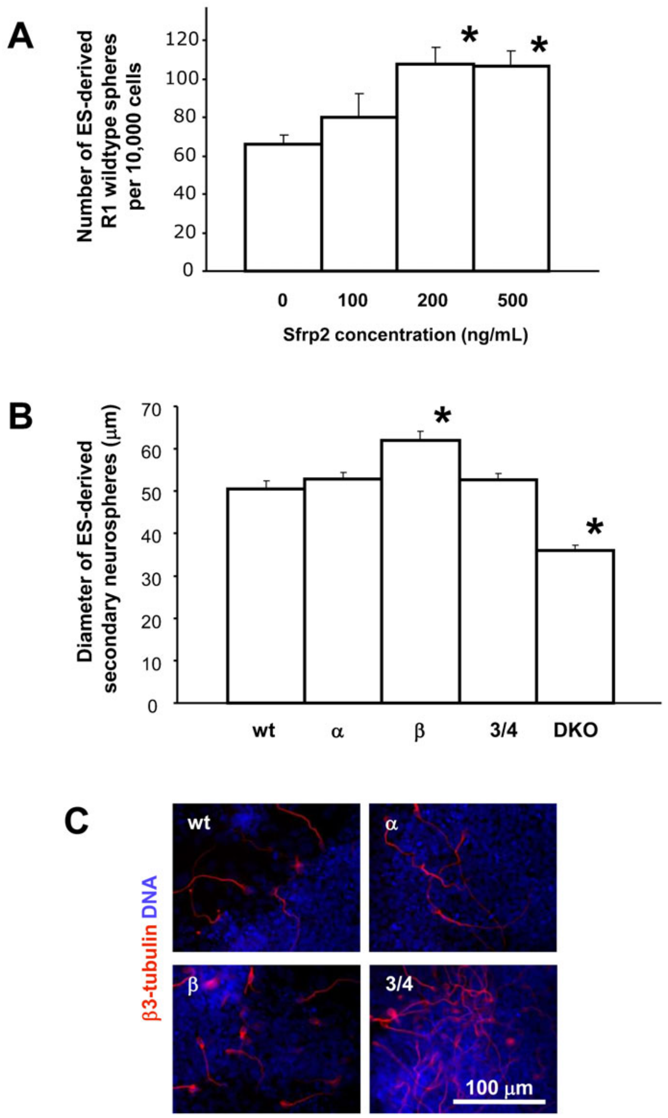

Fig. S2. (**A**) The Wnt inhibitor Sfrp-2 significantly increases the number of ES-cell-derived primitive neurospheres. Data presented as mean±S.E.M. One-way ANOVA showed a significant difference for Sfrp2 dose; $F_{3,20}=5.293$, $P<0.05$. Holm–Sidak t-tests showed significant increases compared to no Sfrp2 control: 200 ng/mL Sfrp2, $t_{10}=3.314$, *$P<0.05$; 500 ng/mL Sfrp2, $t_{10}=3.222$, *$P<0.05$). (**B**) When the size of secondary passage neurospheres generated from ES cell lines bearing different genetic levels of GSK-3 is measured, they show a biphasic response to the dose of GSK-3. Homozygous loss of GSK-3β alleles leads to a significant increase in sphere size, while further loss of GSK-3 alleles as in the 3-quarter (3/4) mutant and the double knockout (DKO) leads to a diminution in sphere size. Data presented as mean±S.E.M. One-way ANOVA showed a significant difference for genotype, $F_{4,499}=16.680$. Holm–Sidak t-tests showed significant differences for the homozygous β mutant, $t_{229}=4.725$, *$P<0.05$; and the DKO mutant, $t_{152}=4.356$, *$P<0.05$, compared to wildtype control. (**C**) Neurospheres from wildtype and partial GSK-3 mutant lines, but not the DKO line, are able to differentiate into neurons (red, β3-tubulin; blue, DAPI). Scale bar: 100 µm.
